# Delayed sowing limits grain number per spike in wheat by restricting young spike differentiation through reduced photothermal resources

**DOI:** 10.3389/fpls.2026.1862259

**Published:** 2026-06-18

**Authors:** Wenqiang Tian, Jiayu Wang, Shan Yu, Alanu Akesumu, Zhilin Zhang, Renyi Dong, Jianhua Wang, Shubing Shi, Jinshan Zhang

**Affiliations:** 1 College of Agronomy, Xinjiang Agricultural University, Urumqi, China; 2 Xinjiang Uygur Autonomous Region Agricultural Technology Extension Station, Urumqi, China; 3 Department of Agronomy, Tumushuke Vocational and Technical College, Tumushuke, China; 4 College of Agronomy and Biotechnology, China Agricultural University, Beijing, China

**Keywords:** delayed sowing, grain number per spike, light and heat resources, winter wheat, young spike differentiation

## Abstract

Delayed sowing has become increasingly common in wheat production, yet the key developmental processes underlying the reduction in grain number per spike remain poorly understood, particularly the relationships among photothermal resources, young spike differentiation, and spike formation. In this study, a two-year field experiment was conducted with five delayed sowing-date treatments at 15-day intervals, and accumulated temperature, actual sunshine duration, and total solar radiation during young spike differentiation were integrated with the overall morphology of young spikes, spikelet/floret primordium differentiation, mature spike structure, and grain number per spike for analysis. The results showed that, as sowing was delayed, young spike differentiation was generally postponed and its duration was markedly shortened, while accumulated temperature, actual sunshine duration, and total solar radiation continuously declined. These changes were accompanied by restricted development of young spike morphology (length, width, and lateral width), reduced differentiation of spikelet and floret primordia, impaired formation of mature spike structure (length, width, and lateral width), and decreases in grain number per spike, including spikelet number, floret number, and fertility, as well as grain weight per spike. These stress effects became stronger with further delays in sowing. Further integrated analysis showed that the decline in grain number per spike under delayed sowing was consistent with a continuous association pathway of “reduced photothermal resources → restricted young spike development → impaired spike formation → decreased grain number per spike.” Among these processes, young spike development was the key link connecting environmental changes with grain number per spike formation, and the decrease in actual sunshine duration showed the strongest statistical association with the reduction in grain number per spike. These findings indicate that the restricted formation of grain number per spike under delayed sowing is closely associated with changes in young spike development and mature spike formation, and they provide a basis for stabilizing wheat yield management under delayed sowing conditions.

## 1 Introduction

Wheat is one of the most important staple crops worldwide, providing about 20% of the daily caloric intake for humans and supporting the livelihoods of billions of people (Chen et al., 2025; Luo et al., 2019). In recent years, a combination of factors, including the increasing frequency of extreme weather events, constraints imposed by preceding crops, and adjustments in agricultural practices, has gradually narrowed the optimal sowing window for wheat. As a result, delayed sowing has become increasingly common (Sun et al., 2024; Tian et al., 2025a).

In double-cropping regions, heavy rainfall during the autumn sowing season often leads to excessive soil moisture, resulting in seed suffocation and seedling rot. Consequently, farmers are compelled to delay sowing to ensure safe emergence conditions (Liu et al., 2022; Rezaie et al., 2022). In areas following a two-year, three-crop rotation system, sowing is often delayed to prolong the growth period of the preceding crops and maximize economic returns (Yang et al., 2025; Zhang et al., 2025b). Similarly, in single-cropping systems, continuous cultivation of cash crops such as cotton and maize contributes to soil degradation, whereas introducing wheat into crop rotations can improve soil fertility. However, the late harvest of preceding crops substantially delays wheat sowing, and the relatively lower yield potential of spring wheat further promotes the adoption of late-sown winter wheat in these areas (Shah et al., 2020; Tian et al., 2025a). Consequently, the expansion of delayed sowing practices, together with the resulting mismatch between wheat growth and optimal environmental conditions, has become a major threat to global food security.

Delayed sowing results in wheat being sown outside the optimal sowing window, leading to an ecological mismatch between crop growth and development and the availability of photothermal resources. This mismatch generates a series of physiological constraints that persist throughout the growth period and ultimately limit the formation of spike number, grain number per spike, and thousand-grain weight, resulting in yield loss (Liu et al., 2022; Tian et al., 2026). Late sowing shortens the pre-winter growth period of wheat and, in combination with unfavorable growth conditions, such as reduced accumulated temperature, actual sunshine duration, and solar radiation, suppresses seed germination and tiller formation. This leads to lower emergence rates, a higher proportion of weak seedlings, and increased overwintering mortality, thereby restricting spike formation from main stems and tillers and ultimately resulting in insufficient spike number (Jarecki, 2024; Kumar et al., 2022; Villegas et al., 2016).

Furthermore, delayed sowing causes wheat development to coincide with rapidly rising temperatures in early spring, accelerating phenological progression and shortening the vegetative growth period. This reduces assimilate accumulation and intensifies competition between vegetative and reproductive growth, thereby impairing young spike development, reducing primordium differentiation, and increasing degeneration rates. These effects collectively restrict grain number per spike formation (Liu et al., 2022; Tian et al., 2025a). In addition, delayed sowing leads to growth retardation and poor development, shortens the grain-filling period, inhibits efficient assimilate transport and allocation to grains, and markedly increases the risk of high-temperature stress during the grain-filling stage, thereby severely threatening the formation and stability of thousand-grain weight (Tian et al., 2026, 2025b). Notably, these adverse effects intensify with further delays in sowing, resulting in progressively greater yield losses (Jarecki, 2024; Klepeckas et al., 2020). Although previous studies have systematically investigated the regulatory mechanisms and compensatory responses of spike number and thousand-grain weight under delayed sowing conditions (Jarecki, 2024; Liu et al., 2022; Luo et al., 2019; Tian et al., 2025a, 2026, 2025b), the mechanisms underlying grain number per spike formation and its contribution to yield loss remain insufficiently understood.

Young spike development is a critical process determining both mature spike formation and grain number per spike in wheat (Gauley et al., 2024). Previous studies have demonstrated from different perspectives that delayed sowing postpones young spike differentiation, shortens the developmental process, causes deviation from optimal light and temperature conditions, leads to ecological mismatch, and ultimately reduces spikelet number, fertile floret number, and grain number per spike (Kumar et al., 2022; Yin et al., 2018). Other studies have separately focused on the effects of temperature, radiation, or shading on reproductive formation in wheat (Abbate et al., 1997; Dong et al., 2019; Gauley et al., 2024; Tian et al., 2025a). However, these studies have mostly remained at the level of explanations based on single environmental factors or endpoint traits, and the following questions remain unclear. First, how changes in accumulated temperature, actual sunshine duration, and total solar radiation caused by delayed sowing are associated within the same developmental response framework remains unclear. Second, whether changes in environmental resources are transmitted to mature spike structure and grain number per spike through early changes in young spike morphological growth and primordium differentiation remains to be determined. Finally, the relative strength of association between different photothermal variables and the decline in grain number per spike under delayed sowing conditions remains unclear. These key issues warrant further in-depth investigation.

Therefore, this study conducted a delayed sowing experiment in the Tacheng region of Xinjiang, China, a typical single-cropping area characterized by seasonal water scarcity and strong winds that hinder the installation of drip irrigation systems and further delay winter wheat sowing. Five delayed sowing-date treatments were established, and accumulated temperature, actual sunshine duration, and total solar radiation during young spike differentiation were continuously observed and subjected to path analysis together with young spike overall morphology, primordium differentiation number, mature spike structure, and grain number per spike. This study aimed to clarify the relationships between the mismatch between measured photothermal resources and young spike development, as well as the formation of spike sink capacity under delayed sowing conditions. It further focused on analyzing the cascade of links among spikelet primordia, floret primordia, and mature spike structure, and on how changes in environmental resources are associated with the decline in grain number per spike. The relative contributions of actual sunshine duration, total solar radiation, and accumulated temperature to grain number per spike formation were compared to identify the key developmental stages responsible for the decline in grain number per spike in late-sown wheat and to determine the environmental indicator most strongly associated among the observed photothermal variables.

## 2 Materials and methods

### 2.1 Site description

The experiment was conducted from September 2021 to August 2023 at the Tacheng Agricultural Science Research Institute (N46°21′, E82°41′), located at an elevation of 415 m. The soil type was sandy loam, and the preceding crop was wheat.

Prior to the experiment, soil samples collected from a depth of 0–20 cm were analyzed, showing a pH of 8.47, organic matter content of 10.37 g kg^–1^, total nitrogen of 0.72 g kg^–1^, available nitrogen of 45.37 mg kg^–1^, available phosphorus of 4.12 mg kg^–1^, and available potassium of 107.9 mg kg^–1^. Diammonium phosphate was applied as a basal fertilizer at a rate of 300 kg hm^–2^ before sowing.

### 2.2 Experimental design

A single-factor randomized complete block design was employed with five sowing dates: September 25 (optimal local sowing date), October 10, October 25, November 9, and November 20, designated as T1, T2, T3, T4, and T5, respectively. The wheat cultivar ‘Xindong 18’, which is widely cultivated in this region and has strong overwintering ability suitable for delayed sowing conditions, was used in this study. Basal irrigation of 750 m^3^ hm^–2^ was applied three days before sowing to ensure adequate soil moisture. Seeds were sown manually in furrows at a rate of 375 kg hm^–2^, with a row spacing of 20 cm and a sowing depth of 3–5 cm.

Each plot measured 2 m × 5 m (10 m^2^) and included three replicates. Each sowing-date treatment corresponded to one independent plot within each block; therefore, each treatment had three independent experimental plots per year. After snowmelt in the second year, seedlings were uniformly thinned within the designated sampling area (middle 10 rows of each plot) to achieve a final planting density of 6.00 × 10^6^ plants hm^–2^. All sampling was conducted independently within each replicate plot, avoiding border rows and the ends of the plot, and focusing on the central measurement area to reduce edge effects and within-plot spatial heterogeneity. All other agronomic practices followed local high-standard management protocols.

### 2.3 Measurement indicators

#### 2.3.1 Meteorological data

Field temperature, actual sunshine duration, and solar radiation were obtained from an SN-QXZ meteorological station installed in the experimental field (Sain Electronics Co., Ltd., Jinan, China). Accumulated temperature, defined as the sum of daily mean temperatures, actual sunshine duration, defined as the sum of daily sunshine hours, and solar radiation, defined as the sum of daily total solar radiation, were calculated for each treatment from the single-ridge stage to the tetrad formation stage of young spike differentiation. Actual sunshine duration refers to the cumulative hours of actual sunshine recorded by the meteorological station and is affected by cloud cover and weather conditions.

#### 2.3.2 Young spike traits

After wheat seedling emergence, five representative main-stem plants were selected from each replicate plot of each sowing-date treatment at 1-day intervals, except during periods of snow cover in winter and spring. Young spikes were dissected as described by Faci et al. (2024). High-definition images were captured using an SZM7045 optical microscope (Sunyu Optics Technology Co., Ltd., Yuyao, China). The developmental stages of young wheat spikes were identified according to the method described by Serrago et al. (2008), including the transition apex stage (W 1.0), when the growth cone was longer than it was wide and appeared as a conical protrusion; the single-ridge stage (W 1.5), when hemispherical ring-shaped protrusions appeared at the base of the growth cone and were arranged as a single ridge; the double-ridge stage (W 2.0), when secondary protrusions appeared in the middle and lower parts of the single ridge and formed a double-ridge structure together with bract primordia; the glume differentiation stage (W 3.0), when glume primordia appeared at the base of spikelet primordia and young spike morphology became more complex; the floret primordium stage (W 4.0), when floret primordia differentiated inside the glumes; the stamen and pistil differentiation stage (W 5.0), when floret primordia differentiated into stamen and pistil primordia; the Waddington scale stage (W 5.5), when the anther connective formed in the middle of the stamen primordia and the anther outline was established; and the tetrad formation stage (W6.0), when pollen mother cells underwent meiosis to form tetrads.

**Figure 1:**
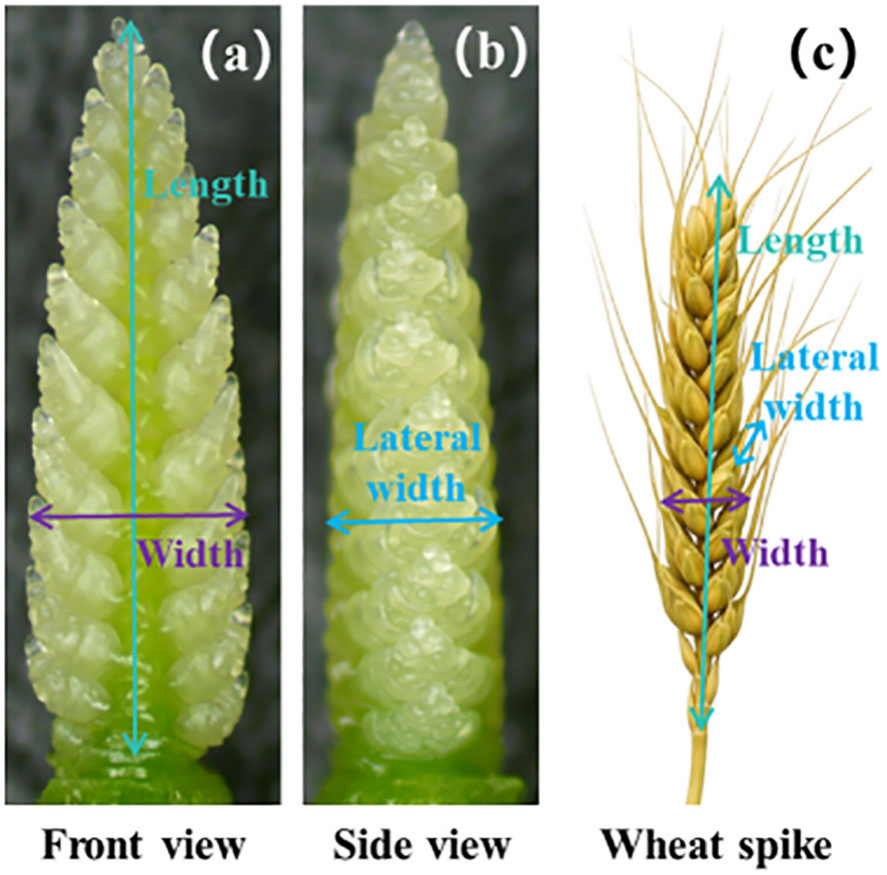
Schematic diagram showing the measurement of spike morphological traits at different developmental stages. **(a, b)** The length, width, and thickness of young spikes were measured using Image-Pro Premier 2024 software. **(c)** Spike length and width of mature spikes were measured using a ruler.

The length, width, and thickness of young spikes (**Figures 1a, b**) were measured using CLRIImage-Pro Premier 2024 software (Media Cybernetics, Bethesda, Maryland, USA). In addition, the number of spikelets and floret primordia was recorded.

#### 2.3.3 Spike traits

After heading, five representative main-stem wheat plants were selected from each replicate plot of each sowing-date treatment. Measurements were conducted every 3 days from heading to flowering and every 5 days from flowering to maturity. Spike length, width, and thickness (**Figure 1c**) were measured using a ruler, and the weight of each individual spike was recorded.

At maturity, five representative main-stem wheat plants were selected from each replicate plot of each sowing-date treatment. The numbers of fertile and sterile spikelets as well as fertile and sterile florets were determined based on final grain set in mature spikes, following the concepts of spikelet fertility and floret fertility described by Sakuma and Schnurbusch (2020) and further applied in wheat fertility studies by AY et al. (2024). Fertile spikelets were defined as spikelets bearing at least one grain, whereas sterile spikelets contained no grains. Fertile florets were defined as florets that successfully developed into grains, whereas sterile florets failed to set grains. Spikelet fertility was calculated as the number of fertile spikelets divided by the total number of spikelets multiplied by 100%, and floret fertility was calculated as the number of fertile florets divided by the total number of florets multiplied by 100%. The total grain weight of each individual spike was also measured.

### 2.4 Statistical analysis

Data were processed using Microsoft Excel 2021 (Microsoft Corp., Redmond, WA, USA). For traits measured on individual plants, values were first averaged within each replicate plot to yield a single observation per plot. Before analysis of variance, all datasets were verified to meet the assumptions of normal distribution using the Shapiro-Wilk test and homogeneity of variance using Levene’s test. Two-way analysis of variance was performed using SPSS 29.0 (IBM Corp., Chicago, IL, USA), with year, sowing date, and their interaction treated as fixed effects to evaluate their effects on the main parameters. When the main effects or interactions were significant, *post hoc* multiple comparisons were performed using Duncan’s new multiple range test at *P* < 0.05. When the year × sowing date interaction was significant, a one-way analysis of variance was conducted for each year to compare differences among sowing date treatments. Overall, the sowing-date effect was the main source of variation in the major parameters. Year effects and year × sowing date interactions were observed only for some indicators, whereas the response direction to delayed sowing remained consistent across the two years. Therefore, the results section presents data from the two years separately and focuses on interpreting the sowing-date effects that were consistent across years.

**Figure 2:**
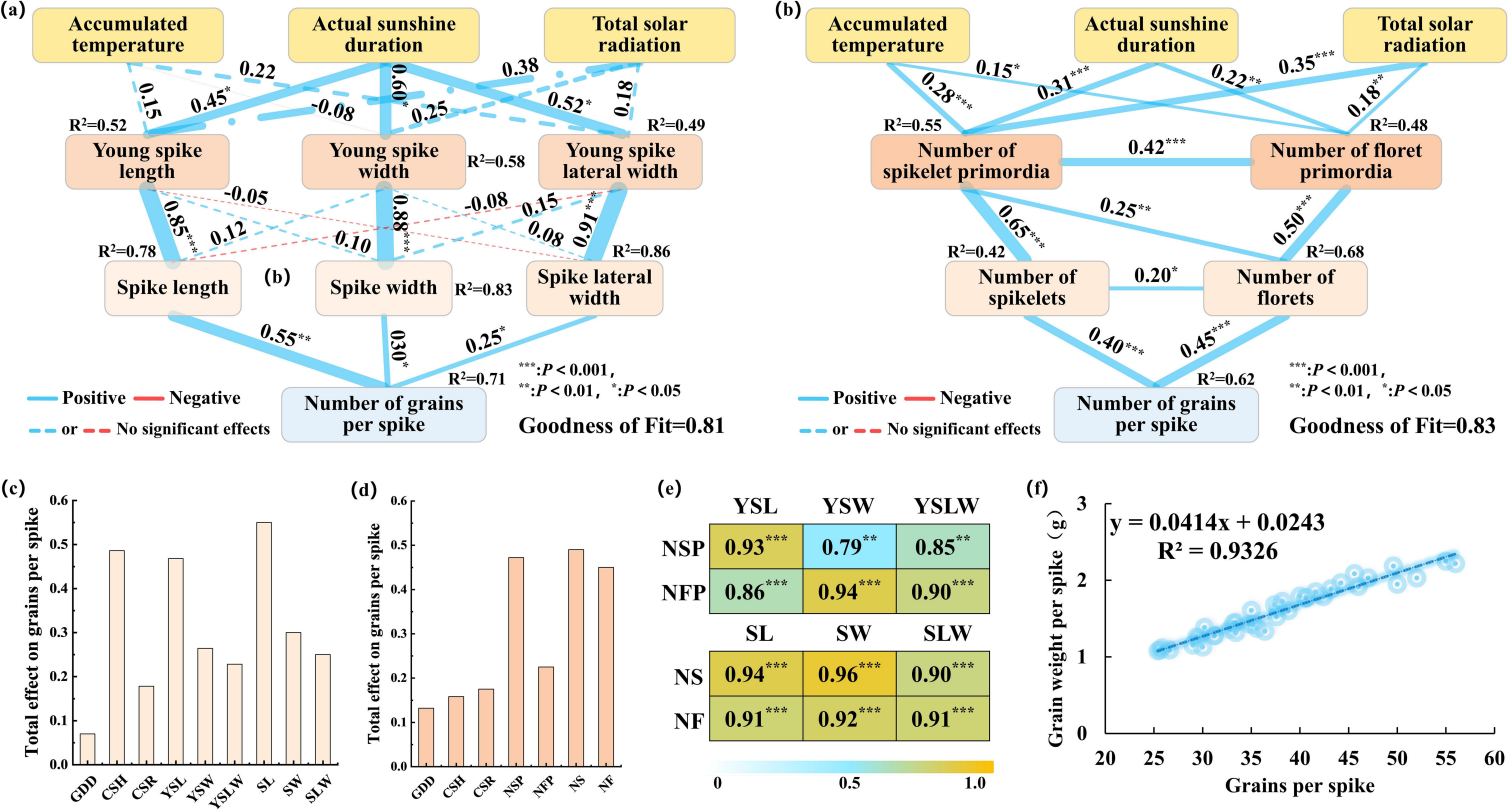
Partial least squares path modeling (PLS-PM), correlation analysis, and simple linear regression analysis. **(a, b)** Positive and negative effects of the related indices in partial least squares path modeling (PLS-PM). Blue arrows indicate positive effects, and red dashed arrows indicate negative effects, while blue or red dashed lines indicate non-significant effects. **(c, d)** Standardized total effects of the related indices, including both direct and indirect contributions. **(e)** Correlation heatmap of the related indices. **(f)** Simple linear regression analysis between grain number per spike and grain weight per spike; each point represents one replicate. R^2^ denotes the proportion of variance explained by the model. ^*^, ^**^, and ^***^ represent significance at *p* < 0.05, *p* < 0.01, and *p* < 0.001, respectively; no asterisk indicates non-significance (*p* > 0.05). Abbreviations are as follows: GDD, CSH, and CSR indicate growing degree days, cumulative sunshine hours, and cumulative solar radiation, respectively; YSL, YSW, and YSLW denote young spike length, young spike width, and young spike lateral width, respectively; SL, SW, and SLW indicate spike length, spike width, and spike lateral width, respectively; NSP and NFP denote number of spikelet primordia and number of floret primordia, respectively; and NS and NF indicate number of spikelets and number of florets, respectively.

Partial least squares path modeling (PLS-PM) was performed using R 4.3.2 (R Core Team, Vienna, Austria). All indicators were standardized using Z-scores before modeling. Based on the research hypotheses, two *a priori* models were constructed. Model I was used to test the morphological formation pathway of “photothermal resources → young spike overall morphology → mature spike overall morphology → grain number per spike.” Model II was used to test the quantitative formation pathway of “photothermal resources → young spike primordium differentiation → mature spike organ number → grain number per spike.” In **Figure 2**, each node was a measured single-indicator construct; therefore, the outer loading was fixed at 1. Model interpretation focused on structural paths, total effects, and the explanatory power of endogenous variables rather than on evaluating Cronbach’s α or average variance extracted (AVE) for multi-indicator latent variables. Path significance was tested using 5,000 bootstrap resamples. Model fit and explanatory ability were evaluated using goodness-of-fit (GoF), R2, and path significance, and multicollinearity was diagnosed using variance inflation factors (VIFs). When VIF values were high, the related indicators were interpreted only as coordinated responses of the same biological process, avoiding interpretation of the path coefficients of highly correlated traits as completely independent effects. Correlations among the morphological and quantitative traits of young spikes and mature spikes were analyzed using Pearson correlation analysis. Simple linear regression analysis was used to examine the relationship between grain number per spike and grain weight per spike, and the normality and homoscedasticity of the regression residuals were tested. Figures were generated using Origin 2024b (OriginLab Corporation, Northampton, MA, USA) and Microsoft PowerPoint (Microsoft Corp., Redmond, WA, USA).

## 3 Results and analysis

### 3.1 Developmental process of young spikes and photothermal conditions

**Figure 3:**
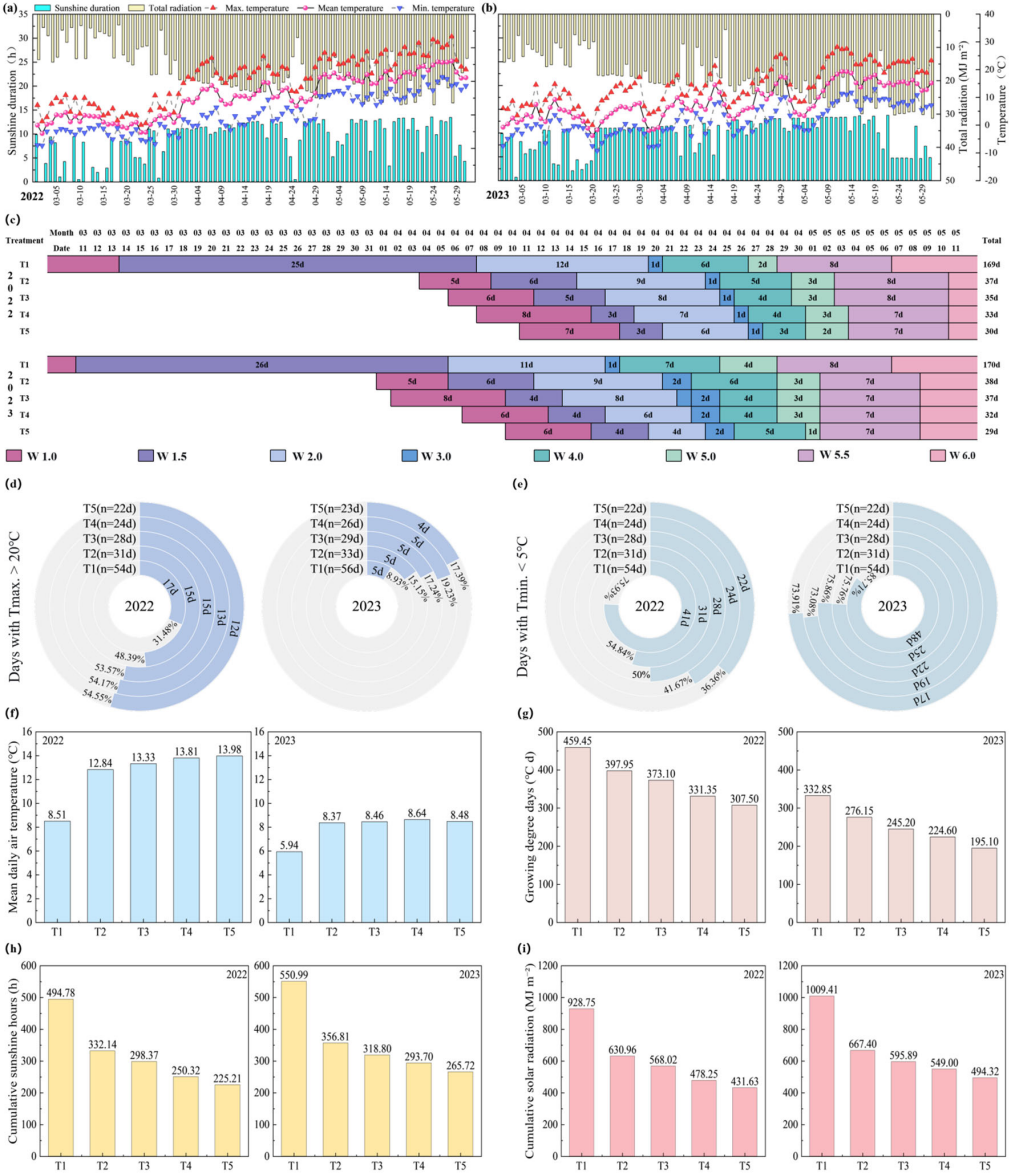
Photothermal conditions and young spike developmental status. **(a, b)** Climatic conditions during wheat growth stages after spring in 2022 **(a)** and 2023 **(b)**. **(c)** Onset and termination dates of the young spike differentiation stage and its duration (days). **(d-i)** Number of days and proportions during young spike development from the single-ridge stage (W 1.5) to the tetrad formation stage under different climatic conditions: **(d)** maximum temperature >20 °C; **(e)** minimum temperature <5 °C; **(f)** mean temperature; **(g)** accumulated temperature; **(h)** actual sunshine duration; and **(i)** total solar radiation. T1, T2, T3, T4, and T5 represent sowing dates of September 25, October 10, October 25, November 9, and November 20, respectively.

As shown in **Figure 3**, sowing dates significantly affected both the young spike differentiation process and the associated photothermal environment, with consistent trends observed across the two years. Delayed sowing (T1-T5) markedly postponed young spike development and shortened the duration of each developmental stage, particularly the single-ridge and double-ridge stages (**Figure 3c**).

Meanwhile, the photothermal conditions corresponding to the young spike development stage changed markedly. As sowing was delayed, the number and proportion of high-temperature days above 20 °C encountered during young spike development generally increased, and the mean temperature also increased. In contrast, accumulated temperature, actual sunshine duration, and total solar radiation showed overall decreasing trends. The reductions were most pronounced in T5, which decreased by 36.56%, 53.06%, and 52.23%, respectively, compared with T1 based on the two-year average. However, the early sowing treatments experienced a higher frequency of low-temperature days below 5 °C (**Figures 3d-i**).

Interannual comparison showed that heat resources during the young spike differentiation period, including the frequency of daily maximum temperature above 20 °C, mean temperature, and accumulated temperature, were generally higher in 2022 than in 2023. In contrast, the frequency of daily minimum temperature below 5 °C was lower. In contrast, the measured light-related variables during the young spike differentiation period, namely actual sunshine duration and total solar radiation, were generally lower in 2022 than in 2023. This interannual difference provides background information for interpreting the relative associations of heat resources and measured light-related variables. Although heat conditions were more abundant in 2022, young spike formation and grain number per spike were still lower than those in 2023.

However, because other environmental and developmental factors may also have varied across years, this result suggests only that decreases in actual sunshine duration and solar radiation accumulation under delayed sowing conditions were closely associated with restricted normal development and differentiation of young spikes.

### 3.2 Growth and differentiation of young spikes

**Figure 4:**
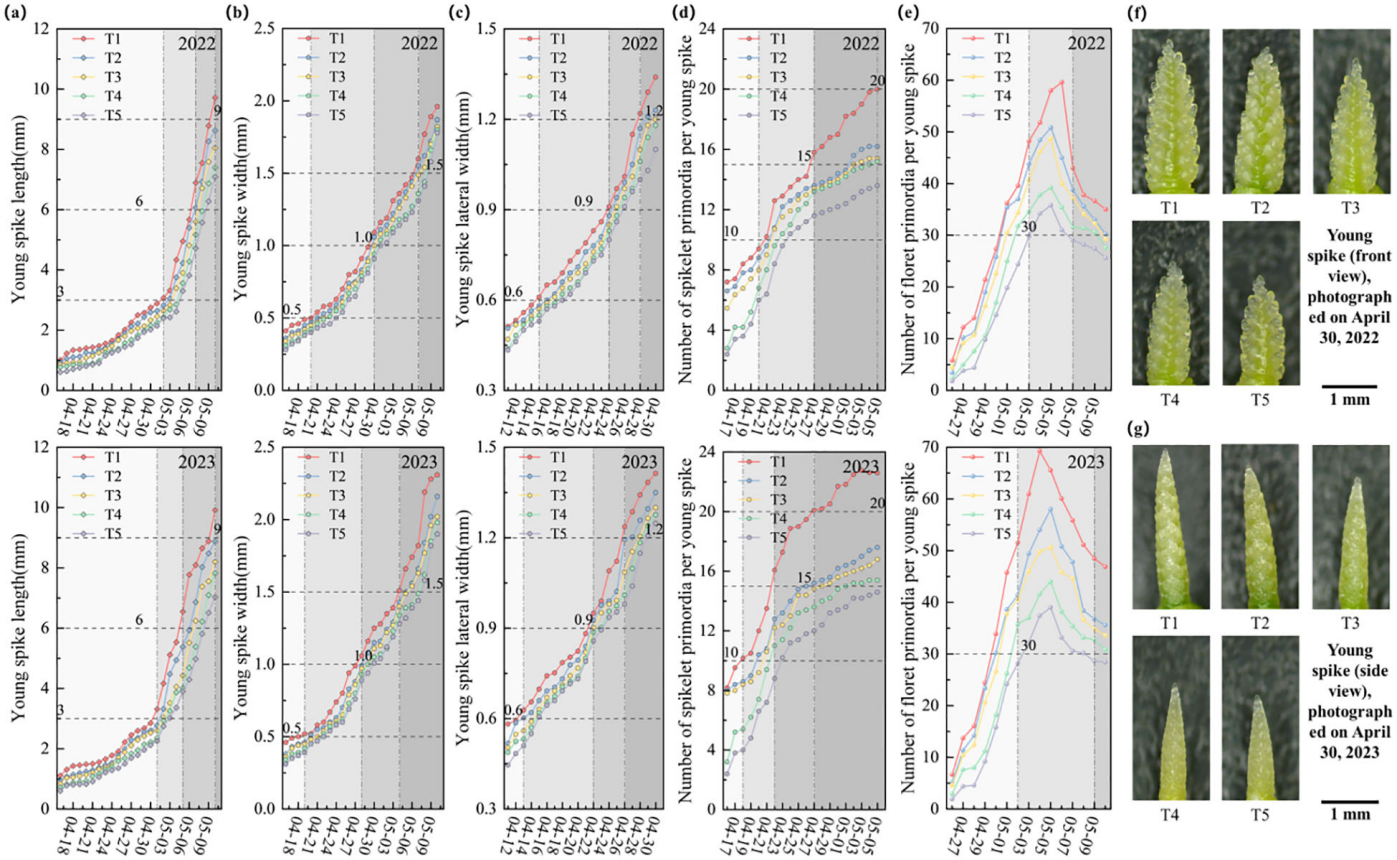
Structure and morphological traits of young spikes. **(a-e)** Changes in young spike morphology and development across stages: **(a)** length, **(b)** width, **(c)** lateral width, **(d)** number of spikelet primordia, and **(e)** number of floret primordia. The horizontal dashed lines indicate the corresponding y-axis values, and the vertical dashed lines represent the corresponding dates of occurrence. **(f, g)** Representative images of young wheat spikes: **(f)** front view and **(g)** side view. T1, T2, T3, T4, and T5 represent sowing dates of September 25, October 10, October 25, November 9, and November 20, respectively.

As shown in **Figure 4**, sowing date significantly influenced the morphological development of young spikes and the dynamics of spikelet and floret primordia differentiation, with consistent patterns observed across both years. During development, the overall morphological indices of young spikes increased continuously, accompanied by a steady rise in the number of spikelet primordia. In contrast, the number of floret primordia initially increased and subsequently declined.

Delayed sowing postponed the growth and development of young spikes and significantly reduced both their overall morphology and differentiation capacity. Among treatments, T1 exhibited the fastest growth and the most favorable morphological development, with the highest spike length, width, and lateral width throughout the developmental process, as well as the greatest numbers of differentiated spikelet and floret primordia. In contrast, T5 showed the most pronounced reductions, with peak values decreasing by 28.09%, 13.67%, 14.91%, 33.80%, and 41.96%, respectively, compared with T1 (two-year average) (**Figures 4a-e**).

Interannual comparisons revealed that young spike morphological development and organ-differentiation capacity were generally lower in 2022 than in 2023, particularly under late-sowing conditions (**Figures 4a-e**). Visual observations (**Figures 4f, g**) further showed that early sowing produced fuller and more developed young spikes with higher differentiation levels, whereas delayed sowing resulted in slender spikes with insufficient organ differentiation.

This indicates that, under delayed sowing conditions, the growth and differentiation levels of young spikes decreased, which was unfavorable for spike formation.

### 3.3 Growth and differentiation of wheat spikes

**Figure 5:**
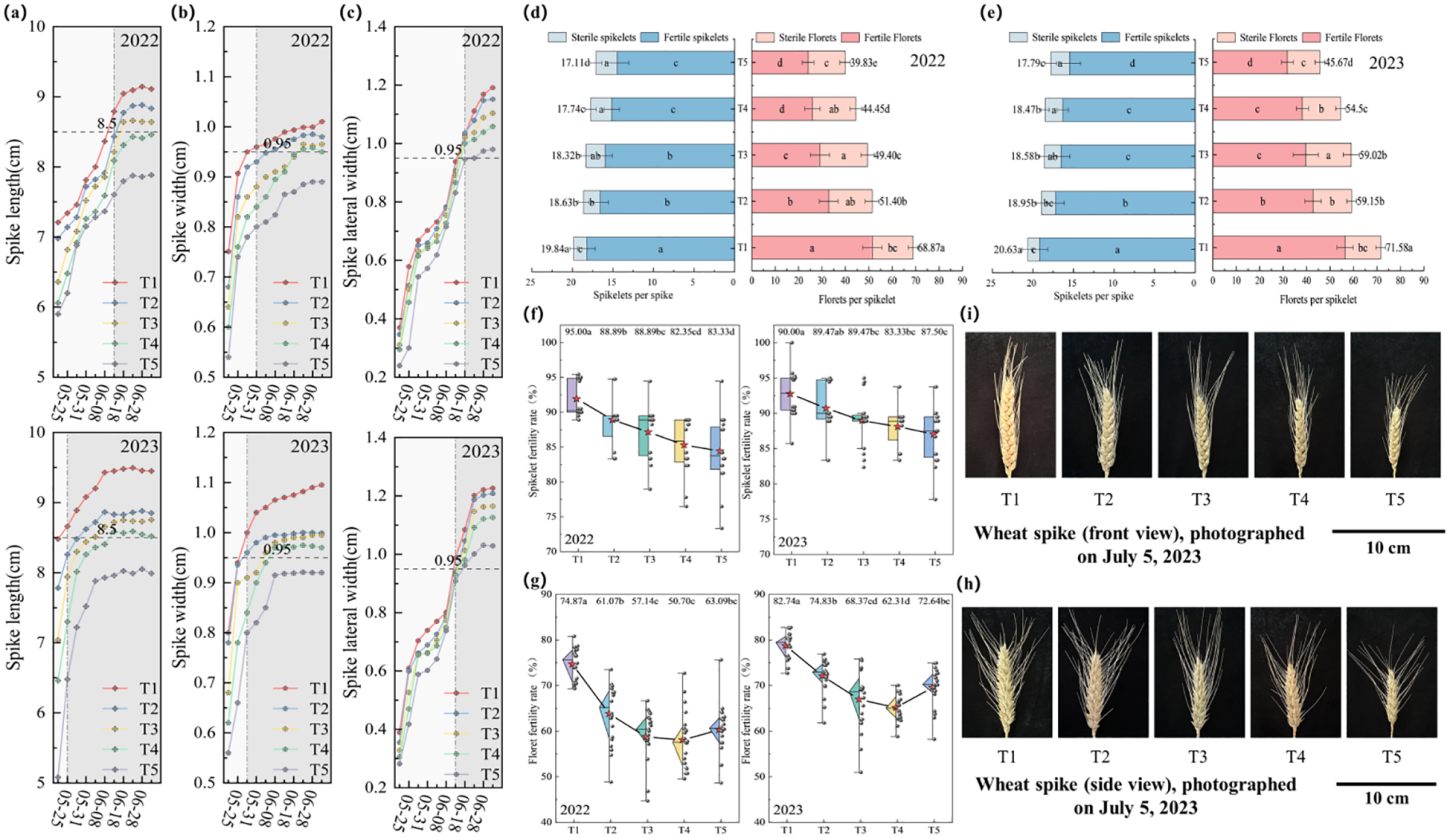
Structure and morphological traits of mature wheat spikes. **(a-c)** Changes in spike morphology during different stages of wheat spike growth: **(a)** length, **(b)** width, and **(c)** lateral width. The horizontal dashed lines indicate the corresponding y-axis values, and the vertical dashed lines represent the corresponding occurrence dates. **(d, e)** Composition of spikelet and floret numbers in wheat spikes. Error bars represent standard errors, and different letters indicate significant differences based on the mean values of replicate plots (*P* < 0.05). **(f, g)** Grain-setting rates of spikelets and florets in wheat spikes. Vertical lines indicate data ranges; black circles represent individual data points, and red stars indicate mean values. Different letters indicate significant differences based on the mean values of replicate plots (*P* < 0.05). **(h, i)** Representative images of mature wheat spikes: **(h)** side view and **(i)** front view. T1, T2, T3, T4, and T5 represent sowing dates of September 25, October 10, October 25, November 9, and November 20, respectively.

As shown in **Figure 5**, sowing date significantly affected the morphological development of wheat spikes, as well as the number of spikelets, florets, and grains, with consistent trends observed across both years.

As wheat spike development progressed, the overall morphological indices continued to increase; however, spike growth was delayed as sowing dates were postponed. Overall, these indices decreased with delayed sowing dates. T1 exhibited the fastest growth, with the greatest mature spike length, width, and lateral width throughout the development period, whereas T5 showed the most pronounced decline. Peak values of all indicators in T5 were reduced by 14.47%, 14.02%, and 16.92%, respectively, compared with T1 (**Figures 5a-c**).

Meanwhile, as sowing was delayed, the number of fertile spikelets and florets decreased, along with a decline in spikelet grain-setting rate. The number of sterile florets initially increased and then decreased, whereas the floret grain-setting rate showed a decreasing-increasing trend. Overall, the total numbers of spikelets and florets were reduced under delayed sowing, with T5 showing decreases of 13.76% and 39.12%, respectively, compared with T1 (**Figures 5d-g**).

Further analysis confirmed that the grain-setting rate of spikelets was generally higher under early sowing but declined under delayed sowing. Interannual comparisons revealed that spike morphological and differentiation indices in 2022 were lower than those in 2023, with more pronounced reductions under late sowing treatments (**Figures 5a-g**).

Additionally, early-sown wheat produced larger, fuller spikes with stronger differentiation capacity, whereas delayed sowing resulted in slender, poorly developed spikes with reduced grain formation (**Figures 5h, i**). This indicates that, under delayed sowing conditions, the growth and differentiation of spikes decreased, which was unfavorable for spike fertility and resulted in insufficient grain number per spike.

### 3.4 Wheat spike weight and grain number

**Figure 6:**
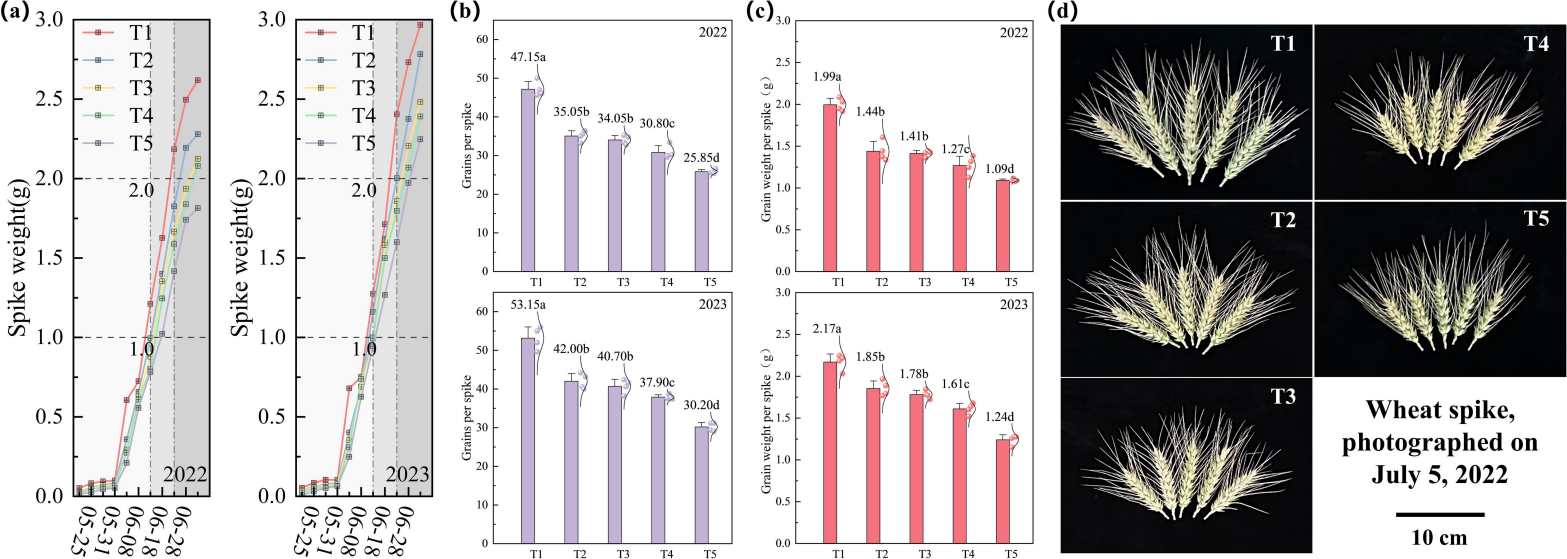
Weight and grain number traits of wheat spikes. **(a)** Changes in single-spike weight. The horizontal dashed lines indicate the corresponding y-axis values, and the vertical dashed lines represent the corresponding occurrence dates. **(b, c)** Grain number per spike and grain weight per spike, respectively. Vertical lines indicate the data range. Circles represent individual data points, and horizontal lines represent error bars. Different letters indicate significant differences based on the mean values of replicate plots (*P* < 0.05). **(d)** Representative image of mature wheat spikes. T1, T2, T3, T4, and T5 represent sowing dates of September 25, October 10, October 25, November 9, and November 20, respectively.

As shown in **Figure 6**, sowing date significantly influenced grain formation in wheat spikes and the final grain number per spike and grain weight, with consistent trends observed across both years.

Spike weight increased progressively during development; however, delayed sowing slowed this increase and reduced overall spike weight. T1 exhibited the fastest accumulation of spike weight and the highest values throughout development, whereas T5 showed the greatest reduction, with peak spike weight decreasing by 28.29% compared with T1 (**Figure 6a**).

Both grain number per spike and grain weight per spike decreased significantly under delayed sowing, with T5 showing reductions of 44.12% and 44.00%, respectively, compared with T1 (**Figures 6b, c**). Interannual comparisons revealed that spike weight, grain number per spike, and grain weight per spike were generally lower in 2022 than in 2023, with larger reductions under late sowing conditions (**Figures 6a-c**). Visual observations (**Figure 6d**) further showed that spikes of early-sown wheat were larger and had fuller grains, whereas spikes of late-sown wheat were smaller and poorly filled.

This indicates that, under delayed sowing conditions, grain number per spike formation and grain filling levels decreased, as reflected by reductions in grain number per spike and grain weight per spike.

### 3.5 Mechanisms underlying the effects of delayed sowing on grain number

To comprehensively analyze the cascade associations among changes in photothermal resources, young spike development, mature spike development, and grain number per spike under different delayed sowing conditions, partial least squares path modeling (PLS-PM), correlation analysis, and simple linear regression were performed (**Figure 2**).

The PLS-PM model supported the existence of a hierarchical association pathway of “photothermal conditions → young spike development → spike formation → grain number per spike” (**Figures 2a, b**). Photothermal conditions, including accumulated temperature, actual sunshine duration, and total solar radiation, showed positive associations with both the overall morphology of young spikes, including length, width, and lateral width, and young spike differentiation capacity, represented by the numbers of spikelet and floret primordia, as indicated by the blue arrows. Young spike development indicators were further significantly and positively associated with spike morphology, including length, width, and lateral width, as well as the numbers of spikelets and florets. Ultimately, spike traits showed a very strong positive direct association with grain number per spike.

Total-effect analysis showed that photothermal conditions, young spike-related traits, and spike-related traits each contributed strongly to grain number per spike. Spike morphological formation and young spike development were key intermediate links connecting photothermal conditions with grain number per spike formation (**Figures 2c, d**). At different hierarchical levels, the factors with the greatest effects on grain number per spike were actual sunshine duration at the photothermal level, young spike length at the young spike level, and spike length at the spike level (**Figure 2c**). In the pathway involving young spike primordia and organ number, total solar radiation, the number of spikelet primordia in young spikes, and the number of spikelets per spike also showed relatively high total effects (**Figure 2d**).

Correlation analysis further showed that the overall morphology of young spikes was generally significantly and positively correlated with young spike differentiation number, and that the overall morphology of spikes was generally significantly and positively correlated with the components of grain number per spike (**Figure 2e**). Meanwhile, grain number per spike had a highly significant positive linear relationship with grain weight per spike (**Figure 2f**), indicating that grain number per spike and grain weight accumulation changed synchronously.

Overall, delayed sowing was associated with the pathway of “reduced photothermal resources → restricted young spike development → impaired spike formation → decreased grain number per spike,” accompanied by a significant reduction in grain weight per spike. In this association pathway, the three photothermal factors differed in their positive associations with young spike development: actual sunshine duration had the greatest total effect, followed by total solar radiation, whereas accumulated temperature had a relatively smaller effect. This indicates that, among the observed photothermal variables, actual sunshine duration was most strongly associated with the reduction in grain number per spike in late-sown wheat. In addition, this result should not be interpreted as an independent causal effect after excluding other covarying environmental and developmental factors.

## 4 Discussion

### 4.1 “Ecological mismatch” between photothermal resources and young spike development under delayed sowing conditions

Late sowing shifts young spike development out of the optimal window for light and temperature. As a result, wheat experiences low-temperature and low-light stress during vegetative growth and early young spike differentiation before winter. Meanwhile, the combined effects of rapid post-spring warming and high light intensity accelerate development, thereby shortening the period of young spike differentiation (Liu et al., 2022; Tian et al., 2026). Previous studies have shown that the “pre-inhibition and post-promotion” stress associated with late sowing becomes increasingly pronounced as sowing is further delayed (Jarecki, 2024; Klepeckas et al., 2020).

Consistent with earlier findings (Gauley et al., 2024; Kumar et al., 2022), this study shows that delayed sowing delays overall spike differentiation and severely compresses the critical developmental period, exposing plants to unfavorable photothermal conditions (**Figure 3**). In contrast, optimal sowing ensures synchrony between crop developmental demand and environmental supply, allowing wheat to fully utilize photothermal resources while minimizing exposure to extreme weather events (Jarecki, 2024; Liu et al., 2022; Sun et al., 2024).

However, this study further found that, as sowing was delayed, the actual sunshine duration, total solar radiation, and accumulated temperature required for young spike development gradually decreased, whereas the risk of exposure to high-temperature stress above 20 °C increased (**Figure 3**), which was closely associated with restricted young spike development. These combined effects constrained the development of young spikes. Therefore, late sowing created a temporal and spatial mismatch between developmental requirements and resource availability. This “ecological mismatch” represents a key risk factor for grain number per spike establishment and yield formation.

Association of Reduced Photothermal Resources with Morphological Formation and Differentiation Number of Young Spikes

Late sowing directly reduces the availability of photothermal resources during the young spike developmental stage, thereby limiting cell division and organ differentiation (Abbate et al., 1997; Gauley et al., 2024; Weng et al., 2025). Light and solar radiation are the primary energy sources driving spike differentiation; deficiencies suppress meristem activity and reduce the formation of spikelet and floret primordia (Fischer, 1985; González et al., 2005), consistent with our findings.

In addition, our study revealed that the overall morphological growth of young spikes (length, width, and lateral width) declined significantly with reduced actual sunshine duration and solar radiation (**Figure 4**). Thermal limitations further constrain the development of young spikes, as insufficient accumulated temperature leads to incomplete differentiation (Lin et al., 2023; Xu et al., 2023). In this study, delayed sowing significantly reduced the accumulated temperature during young spike development, and both overall morphology and the numbers of spikelet and floret primordia showed concurrent declines (**Figure 4**).

Both high- and low-temperature stresses negatively affect young spike development. High temperatures accelerate the developmental process and lead to incomplete differentiation, whereas low temperatures disrupt the normal development of spikelet and floret primordia, increasing sterility rates and damaging spike cell structure (Jacott and Boden, 2020; Lin et al., 2023; Prieto et al., 2020; Wang et al., 2024). In this study, delayed sowing increased the frequency of high-temperature events during young spike differentiation while reducing the occurrence of low-temperature events; nevertheless, spike development continued to deteriorate (**Figures 4d-f**).

This phenomenon may indicate that the decline in young spike development under delayed sowing conditions was not only related to the intensity of a single temperature stress, but may also have been associated with deviation of the young spike development process from the optimal temperature window. Thus, reduced photothermal resources were closely associated with decreases in the number of young spike morphs formed and differentiated, and may further affect the establishment of grain number per spike and yield formation in late-sown wheat.

### 4.2 Cascade association pathway of grain number per spike formation under late-sowing stress

The effects of late-sowing stress in wheat are not limited to young spike formation and development under restricted photothermal resources; rather, they extend to subsequent growth and ultimately influence yield potential (Gauley et al., 2024; Kumar et al., 2022; Lin et al., 2023). Previous studies have demonstrated a close quantitative relationship between the quality of young spike differentiation and grain number per spike, and incomplete development usually leads to reductions in the numbers of spikelets and florets (Jarecki, 2024; Wang et al., 2024; Weng et al., 2025).

Building on these findings, this study offers a new insight: extending this relationship from a simple correlation to an interpretable cascade association framework. Under delayed sowing conditions, the resource environment during young spike differentiation changed and was accompanied by restricted expansion of young spike length, width, and lateral width, as well as limited differentiation of young spike primordia. This was subsequently reflected in reduced structural capacity of mature spikes and was ultimately associated with synchronous decreases in grain number per spike and grain weight per spike (**Figures 2e, f**, **4**). This also indicates that grain number per spike loss under delayed sowing conditions may not occur only through floret abortion at flowering or assimilate transport during grain filling, but may already be manifested during early young spike differentiation as “insufficient morphological space → reduced young spike primordium pool → decreased spike sink capacity.”

Therefore, the young spike development stage may be the most sensitive window for establishing grain number per spike under late-sowing stress and a period deserving priority attention in subsequent agronomic interventions. This has also been confirmed by Gauley et al. (2024) and He et al. (2025).

Notably, compared with previous studies that mainly focused on young spike differentiation number or final grain number per spike (Gauley et al., 2024; He et al., 2025; Yin et al., 2018), this study further distinguished the differences in the contributions of young spike morphology and mature spike morphology to grain number per spike. Young spike length and spike length were identified as the morphological indicators most closely related to grain number per spike (**Figures 2c, d**), suggesting that maintaining their longitudinal elongation capacity may be a feasible agronomic management direction for alleviating yield loss under delayed sowing.

However, previous studies have shown that floret abortion and environmental factors during flowering are critical for grain number per spike formation under delayed sowing conditions, and that the grain filling process is also correspondingly restricted, thereby limiting final yield (Tian et al., 2026; Yin et al., 2018; Zhang et al., 2025a). The cascade association pathway in this study involved the flowering and grain-filling processes only to a limited extent, with only dynamic changes in spike weight examined (**Figure 6**). Therefore, future studies should further integrate changes in floret fertility during flowering and the grain formation process during grain filling to improve the whole-growth-period explanatory framework for yield formation under delayed sowing conditions.

However, it should be emphasized that the pathway proposed in this study was mainly based on field phenotypic observations, correlation analysis, and PLS-PM inference. In sowing-date experiments, factors such as calendar date, astronomical daylength, actual sunshine duration, radiation, accumulated temperature, plant age, developmental duration, and source-sink status cannot be completely separated. In addition, photosynthetic activity, carbohydrate availability, biomass allocation, nitrogen status, hormone dynamics, and gene expression were not directly measured in this study. Therefore, this pathway should be understood as an inferential association framework rather than an experimentally proven mechanistic chain or causal evidence for a single environmental factor.

### 4.3 Relative contributions of photothermal factors to young spike development and the dominant role of actual sunshine duration

Wheat young spike differentiation depends on adequate photothermal resources to sustain cell division and primordium differentiation, forming the basis for spike morphogenesis and yield formation (Dong et al., 2019; Serrago et al., 2008; Villegas et al., 2016). Previous studies have shown that accumulated temperature, sunshine duration, and total radiation are all key environmental factors regulating wheat growth, development, and yield formation (Lin et al., 2023; Villegas et al., 2016; Yu et al., 2025).However, existing studies on delayed sowing have mainly emphasized insufficient accumulated temperature or temperature stress effects, and thus often regarded heat resources as important factors limiting spike formation in late-sown wheat (Chen et al., 2024; Jarecki, 2024; Lin et al., 2023; Villegas et al., 2016; Yu et al., 2023).

By simultaneously incorporating accumulated temperature, actual sunshine duration, and total solar radiation, this study observed that light resources, including sunshine duration and total radiation, had stronger positive associations with young spike development and spike formation than heat resources, represented by accumulated temperature (**Figure 2**). The natural interannual differences in photothermal conditions provided background information for this statistical association: heat resources in 2022, including ≥0 °C accumulated temperature and the number of days with daily maximum temperature above 20 °C, were generally greater than those in 2023, whereas measured light-related variables, including actual sunshine duration and total solar radiation, were lower than those in 2023 (**Figure 3**). However, all indicators of young spike development and spike formation in 2023 were significantly higher than those in 2022 (**Figures 4**–**6**).

Further analysis showed that, among the three photothermal resource factors, sunshine duration had the greatest total effect, followed by total radiation and accumulated temperature. This indicates that, in this dataset, light duration was more strongly associated with young spike differentiation, spike formation, and the reduction in grain number per spike in late-sown wheat, but this does not prove that it was an independent physiological mechanistic factor. In addition, total solar radiation mainly reflects energy intensity, accumulated temperature mainly reflects heat accumulation, whereas actual sunshine duration simultaneously links developmental time, photosynthetic continuity, and potential photoperiodic responses. Therefore, in this experimental design, actual sunshine duration may better characterize the integrated response of young spike morphological formation and grain number per spike formation under delayed sowing conditions (Lin et al., 2023).

Therefore, in yield-stabilization management and cultivation decision-making for late-sown wheat, management strategies should not be limited to heat compensation, but should pay greater attention to matching the effective light duration during the critical period of young spike differentiation with canopy light transmission, sowing-date selection, and cultivar photoperiod sensitivity. Additionally, cultivar-specific sensitivity to light and heat deficits varies (Dong et al., 2014; Serrago et al., 2008), indicating that genotype-dependent responses should be considered. Based on this, future studies should conduct additional validation experiments across different cultivars, including light- and temperature-sensitive and light- and temperature-insensitive types, and across different locations, including high- and low-altitude and high- and low-latitude regions, to clarify differences in cultivar responses to changes in photothermal resources.

In addition, it should be noted that, under the specific planting density used in this study, 6.00 × 10^6^ plants hm^–2,^ the response processes of young spike differentiation and grain number per spike under delayed sowing conditions were mainly explained based on accumulated temperature, actual sunshine duration, and total solar radiation. Microenvironmental factors such as soil moisture, relative humidity, vapor pressure deficit, wind speed, canopy temperature, and photosynthetically active radiation were not monitored simultaneously. Especially under the production conditions of seasonal water shortages and frequent strong winds in Tacheng, Xinjiang, these environmental factors may affect young spike development and grain number per spike. Therefore, the conclusions of this study regarding the relative contributions of photothermal variables and the developmental pathway are limited to the present experimental conditions and the range of observed variables. Future studies need to further validate these findings by integrating continuous multifactor observations with physiological, biochemical, and molecular measurements.

## 5 Conclusion

This study showed that, as sowing was delayed, accumulated temperature, actual sunshine duration, and total solar radiation during the young spike differentiation period decreased, while the young spike differentiation process was postponed and compressed. Meanwhile, young spike morphological formation, differentiation of spikelet and floret primordia, mature spike structure, grain number per spike, and grain weight per spike all decreased.

Integrated analysis showed that the decrease in grain number per spike under delayed sowing conditions was closely associated with restricted young spike development, which can be summarized as a continuous association framework of “reduced photothermal resources → restricted young spike development → impaired spike formation → decreased grain number per spike.” Among the observed photothermal variables, actual sunshine duration showed a relatively strong statistical association with the reduction in grain number per spike.

Notably, the above conclusions are limited by the conditions of uniform seedling establishment after snowmelt and the specific planting density, and may not necessarily apply to conventional field yield responses. Future studies should combine controlled experiments across multiple cultivars, locations, and microenvironmental factors, and integrate post-anthesis grain formation processes to further verify the mechanisms underlying these processes.

## Data Availability

The raw data supporting the conclusions of this article will be made available by the authors, without undue reservation.
